# Assessment of the Severity of Left Anterior Descending Coronary Artery Stenoses by Enhanced Transthoracic Doppler Echocardiography: Validation of a Method Based on the Continuity Equation

**DOI:** 10.3390/diagnostics13152526

**Published:** 2023-07-29

**Authors:** Carlo Caiati, Alessandro Stanca, Mario Erminio Lepera

**Affiliations:** Unit of Cardiovascular Diseases, Department of Interdisciplinary Medicine, University of Bari “Aldo Moro”, 70121 Bari, Italy; carlo.caiati@uniba.it (C.C.); alessandrostanca@gmail.com (A.S.)

**Keywords:** accelerated stenotic flow, transthoracic Doppler echocardiography, coronary artery disease, left circumflex coronary artery athero, right coronary artery athero

## Abstract

Background: To verify whether the severity of coronary stenosis could be non-invasively assessed by enhanced transthoracic coronary echo Doppler in convergent color Doppler mode (E-Doppler TTE) over a wide range of values (from mild to severe). Methods: Color-guided pulsed wave Doppler sampling in the left anterior descending coronary artery (LAD) was performed in 103 diseased LAD segments (corresponding to 94 patients examined) as assessed by quantitative coronary angiography (QCA) or intracoronary ultrasound (IVUS). The E-Doppler TTE examinations consisted of measuring the velocity (vel) at the stenosis site and a reference adjacent segment. Then the continuity equation (C-Eq) was applied to calculate the percent cross-sectional area reduction (%CSA) at the stenosis site. The applied formula was: %CSA = 100 × (1 − [TVIref × 0.5]/TVIs). TVI = the time velocity integral at the stenosis [s] and the reference site [ref], respectively); 0.5 = the correcting factor for a parabolic profile was used only when the % accelerated stenotic flow was >122% (AsF = diastolic peak vel at first site − diastolic peak vel at second site/diastolic peak vel at second site × 100). Results: E-Doppler TTE feasibility was 100%. Doppler and QCA/IVUS-derived %CSA stenosis showed very good agreement over a large range of values (from mild to severe), with no significant bias; the maximum difference between QCA/IVUS and transthoracic Doppler %CSA was mostly around 20% with a few patients exceeding this limit (limits of agreement = −27.53 to 23.5%). The scattering was slightly larger for the non-significant stenoses. The correlation was strong (r = 0.89, *p* < 0.001). Conclusion: E-Doppler TTE is a feasible and reliable method for assessing the severity of LAD stenosis by applying the C-Eq.

## 1. Introduction

The minimal cross-sectional area (CSA) of the stenosis is the most important determinant of stenosis resistance. This is because dynamically, for any given level of flow, the minimal stenotic area appears as a second-order term in both viscous and separation losses [1]. Functional assessment is a superior way to evaluate stenosis, with very important clinical implications. This basically consists of Doppler recording the transtenotic velocity and normalizing it to a velocity reference [2]. Velocity measurements can allow quantitation of the stenosis (% cross-sectional area reduction) by applying the continuity equation (C-Eq) [3,4,5,6,7,8,9]. The principle of continuity of flow is a corollary of the law of conservation of mass, and it states that flow in any portion of a nonbranching tube is equal [10].

However, the transtenotic velocity is not easy to obtain. Some efforts have been made in the past but only with an invasive approach, by recording transtenotic flow velocity by intracoronary Doppler flow wire [3,4,5] or transesophageal Doppler echocardiography [11,12].

Nowadays, E-Doppler Transthoracic Echocardiography (TTE) has enormously increased the feasibility of coronary blood flow Doppler recording in the left main coronary artery (LMCA) and in the whole left anterior descending coronary artery (LAD), allowing routine transtenotic velocity measurement in a clinical setting [13,14,15]. With this approach, the C-Eq can be applied in both significant [12,13] and non-significant stenoses [13]. However, as demonstrated, C-Eq application with this method that measures the peak velocity needs to be corrected by taking into account the different average spatial velocity profiles in the reference (parabolic) and at the stenosis site (more blunted). The maximal transtenotic velocity measured by E-Doppler TTE will, in fact, overestimate the spatial average velocity more with a parabolic than a blunted profile. Therefore, a shape factor correction of 2 must be applied in these cases for the reference parabolic profile. Even if this correction initially seemed to show some utility, it was verified only in a significant stenosis setting [12]. When it was preliminarily extended to non-significant coronary stenosis, the correction became counterproductive. Indeed, a method without correction gave much better results [14].

Therefore, it appears that correction is important only when the velocity profiles are different at the reference and the stenosis sites because a blunting of the profile is occurring at the stenosis site. This blunting effect has to be taken into account only in the cases of functionally significant stenosis, which should create a brisk acceleration of flow at the stenosis site, thus blunting the blood flow profile [16]. One of the established ways to recognize a functionally significant stenosis is through assessment of the pressure drop in the post stenotic region, as assessed by the invasive fractional flow reserve (FFR) in the cath lab [17,18,19,20]. A transtenotic velocity higher than 90 cm/s or a % accelerated stenotic flow (AsF) > 120% in the LAD by E-Doppler TTE successfully predicted a significant hyperemic pressure drop (FFR < 0.8) during cath [21].

In short, the E-Doppler TTE velocity recording in the LAD can also support decision-making as whether to or not to correct the C-Eq. No systematic validation study has yet been made in a large number of patients with either significant or non-significant stenoses using the C-Eq, either corrected or not corrected.

Thus, we hypothesized that C-Eq could be properly applied during E-Doppler TTE based on the transtenotic velocity characteristics of the blood flow profile, as predicted by %AsF. As the gold standard of coronary stenosis severity, we used either intravascular ultrasound (IVUS) for non-significant stenosis or quantitative coronary angiography (QCA) for significant stenosis.

## 2. Materials and Methods

### 2.1. Study Groups

Ninety-four consecutive patients (103 LAD segments) with AsF in the LAD, as detected by E-Doppler TTE, underwent cath and their LAD stenosis was quantified by QCA or IVUS. These patients were consecutive and unselected, thus including those with large body sizes (Table 1). This study was exploratory and did not have a predetermined sample size, but it was estimated to be around 70–80 patients. The study protocol was authorized by the Policlinico di Bari, Bari, IT, Institutional Review Board. All patients gave informed consent to take part in this study.

### 2.2. Color Flow Mapping in the LMA and the Whole LAD

Ultrasound equipment and technologies (see Supplementary Materials: Detailed Methods for details).

Ultrasound setting (see Supplementary Materials: Detailed Methods for details).

### 2.3. LAD Segmentation and Anatomy

The LAD flow was measured by color Doppler at different segments of the LAD, starting from the proximal, then the middle, and finally the distal parts [13,14] (see Supplementary Materials: Detailed Methods for the LAD segmentation and anatomy).

### 2.4. Ultrasound Plane Orientation

The parasternal area was scanned from top to bottom using both conventional and novel methods, and then the apical area, following the same procedure as the B-mode detection of the LMCA [22], but adapted for the Doppler measurement of blood flow along the LAD (see Supplementary Materials: Detailed Methods, for details about the LAD ultrasound plane orientation).

One major advance in plane orientation, apart from the previously described apical views [14], consists of a new parasternal approach for the proximal and mid segments. In short, the color flow of the proximal and mid-LAD was enhanced by positioning the patient in extreme lateral decubitus to move the left lung to the side and expose the heart as much as possible, and then by using the cardiac notch of the left lung, sliding the transducer as far as possible to the left on the left half of the chest while keeping optimal heart sonification. Since the sulcus is much more depressed than the pulmonary conus [23], this shift of the probe to the left shows the artery located in the central part of the sector, with maximal ultrasound energy and a narrower theta angle. In this way, a minimal inferior angulation of the probe is more successful in tangentially transecting the depressed content of the sulcus (both proximal and mid part), achieving a narrower theta angle and thus making it possible to record flow for a longer tract in the same plane. Since the probe is laterally displaced, the diagonal branches are also almost regularly transected and properly insonified by the Doppler.

### 2.5. Heart Rate Lowering Protocol

All patients with heart rate (HR) > 65 bpm underwent the HR lowering protocol, receiving 0.7 mg oral delorazepam followed by 100 mg oral metoprolol. After 30 min, their HR was measured: if it was ≤60 bpm, they were given the E-Doppler TTE scan; if still >60 bpm, they would receive intravenous metoprolol 5 mg over 10 min. In a few cases with persistent HR > 65 bpm, pre-treatment for 3 days with 7.5 mg Ivabradine bis in die was prescribed before the E-Doppler TTE [14].

### 2.6. Echocardiographic Data Analysis

The same operator performed all the Doppler readings before the catheterization. Moreover, the maximum length of the LAD color Doppler flow signal in the proximal mid and distal segment was quantified using calipers [11].

### 2.7. E-Doppler TTE: Pulsed-Wave Doppler Analysis

**Figure 1:**
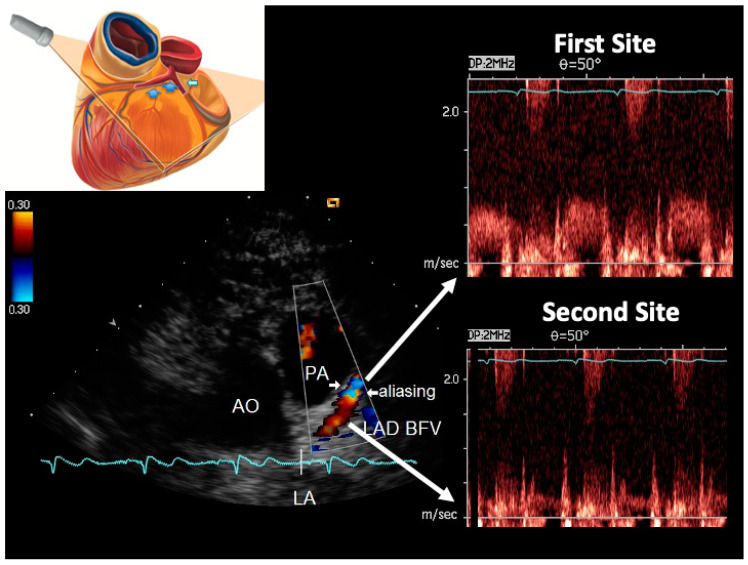
Example of color-guided PW Doppler sampling performed at a stenotic site and a reference site in a case of non-significant stenosis. On the top left is a cartoon that explains the plane orientation adopted to obtain the tomographic view. On the bottom left is the color flow in the LAD with the indicated area of aliasing (arrows). The PW Doppler tracing corresponding respectively to the transtenotic velocity (area of aliasing at the color flow) and the reference velocity (more proximal area of smooth color flow, in red) are reported on the right. The TVI of the spectral diastolic waves obtained from this measurement has to be used in the continuity equation. In this case, the calculation gave a 45% %CSA (the not corrected method was applied as the %ASF is less than 120%). AsF = accelerated stenotic flow; PW = pulsed wave Doppler; TVI = time velocity integral; CSA = % cross-sectional area stenosis; LAD = left anterior descending coronary artery.

**Figure 2:**
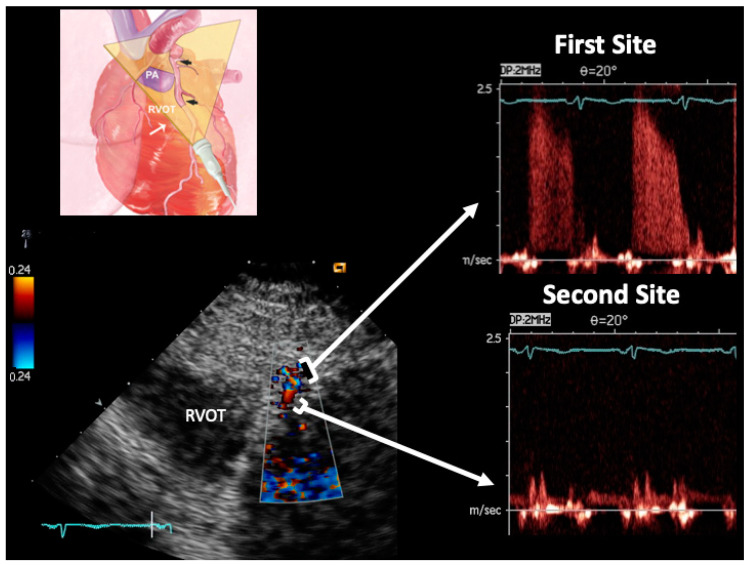
Example of PW Doppler sampling guided by color flow imaging performed at a stenotic site and a reference site in a case of significant stenosis. Same arrangement as Figure 1. In this case, the LAD segment involved is in the mid LAD (see the different plane orientation); the blood flow velocities are much higher at the stenosis site since AsF = 1050%; the %CSA calculated by using TVIs gave a %CSA = 94% (a corrected equation was adopted since the AsF value was >120%). Legends are the same as those in Figure 1.

The peak and time velocity integrals of the diastolic waves were measured at two sites in the LMCA and in each LAD segment (Figure 1 and Figure 2), and the AsF was calculated as the percentage difference between the peak velocity intervals (diastolic peak at first site—diastolic peak at second site/diastolic peak at second site) × 100 [11,13]. The variability and intra and inter-observers’ reproducibility between these two measurements has been previously reported [14,24].

### 2.8. E-Doppler TTE Versus Quantitative Coronary Angiography and IVUS: Doppler Determination of Percentage Area Stenosis

The severity of LAD stenosis was assessed by the means of transthoracic Doppler and applying the C-Eq [12,13]. The C-Eq assumes that the blood flow rate is constant at the stenosis site (Qs) and in the (proximal or distal) adjacent segment without stenosis (Qref), as long as there is no branching between the two. As blood flow is derived from the product of the Doppler curve time velocity integral (TVI) and the cross-sectional area of the vessel, we can obtain

(1) $$Q_{ref}=A_{ref}\times {TVI}_{ref}=A_{s}\times {TVI}_{s}=Q_{s}$$

The percentage area stenosis (%A_s_) can be expressed as

(2) $$\%A_{s}=\left(A_{ref}-A_{s}\right)/A_{ref}\times 100=\left(1-A_{s}/A_{ref}\right)\times 100$$

Rearranging Equations (1) and (2) leads to

(3) $$\%A_{s}=100\times \left(1-{TVI}_{ref}/{TVI}_{s}\right)$$

We also reanalyzed the data using a corrected formula that considers different flow profiles in the reference and stenotic regions: the former is parabolic and the latter flat [4,16]. Doppler, indeed, measures the peak velocity averaged over the cardiac cycle instead of average spatial velocity (the mean velocity), but it is the latter (along with the vessel cross-sectional area) that is required to calculate flow. The average spatial velocity is affected by the velocity profile and, as the blood velocity profile in the parabolically shaped reference segment can be expected to be different from that in the blunted stenotic segment [4,16], the reference TVI (derived from peak and not average spatial velocity) has to be corrected by the shape factor (0.5) for a parabolic profile [12]. This avoids overestimating the reference average spatial flow velocity that consequently leads to underestimated %As.

Equation (3) therefore becomes

(4) $$\%A_{s}=100\times \left(1-\left[{TVI}_{ref}\times 0.5\right]/{TVI}_{s}\right)$$

Intra- and inter-observers Doppler parameters reproducibility has been recently reported [14].

### 2.9. Coronary Angiography and IVUS

The method of performing coronary angiography on patients was determined by the physician’s discretion and based on the patient’s anatomy and clinical condition, using either the trans-femoral or trans-radial route.

The angiographic studies were conducted as routine procedures and were interpreted without knowledge of the TTE Doppler results. A single investigator visually assessed the coronary stenosis based on multiple projections, with a specific focus on checking for the presence of minimal luminal irregularities.

### 2.10. Quantitative Coronary Angiography

#### Significant Stenosis Subgroup

**Figure 3:**
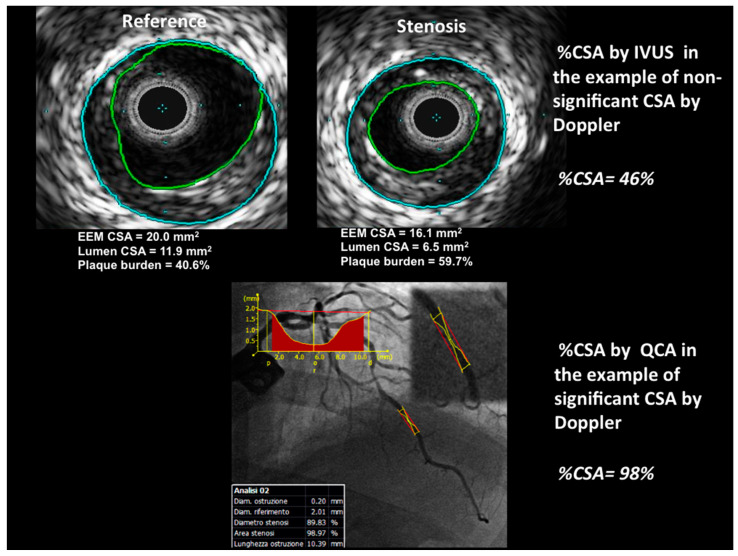
Examples of Doppler stenotic lesions obtained by IVUS (**top**) and QCA (**bottom**). The top image shows the same case as that presented in Figure 1 but evaluated by IVUS: the %CSA measured by IVUS (reference lumen area — stenotic lumen area/reference lumen area × 100) gave a %CSA = 46% equal to that of Doppler CSA; the external elastic layer is also traced. The bottom image shows the QCA evaluation of the same stenosis as that assessed in Figure 2 by Doppler. QCA allowed the automatic tracing of the intimal border and the assessment of the minimal (in red) and reference diameters. The %CSA measurements provided a %CSA of 98% confirming the data of the Doppler study. QCA = quantitative coronary angiography; IVUS = intravascular ultrasounds; CSA = cross-sectional area.

In 44 stenoses (found in 44 patients), the maximum (reference) proximal and distal luminal diameters, as well as the minimum luminal diameter itself, were assessed from digital images using an off-line computerized bidimensional QCA analysis system (QAngio 7.3, Medis medical imaging systems bv, Leiden, The Netherlands). In accordance with the last consensus document published [25], after having chosen the best projection to avoid branch crossing or foreshortening of the lesion to analyze, calibration was conducted using the catheter size (6 French) in order to determine the actual caliper of the coronary artery lumen (Figure 3). The diameter measurements were used to calculate the minimum and reference luminal cross-sectional area (assuming a circular cross-section), after which the percentage cross-sectional area was automatically calculated as:

$$\%A_{s}=\left(A_{ref}-A_{s}\right)/A_{ref}\times 100$$

### 2.11. IVUS Procedure and Analysis of IVUS Data

#### 2.11.1. Non-Significant Stenosis Subgroup

Following the administration of intracoronary nitroglycerine to reverse any arterial spasm, an IVUS examination of the LAD was conducted. The study excluded cases where the luminal diameter, the site, or the extent of vascular disease prevented the insertion of the IVUS catheter. These included situations such as very tight stenosis, diffuse atherosclerosis, major tortuosity, significant myocardial bridges, diffuse or isolated coronary spasms, and wrinkling or invagination of angled segments by the guidewire. These exclusion criteria are aimed at preventing dissection or destabilization of the plaque itself. The digital IVUS catheter (Eagle Eye Platinum, Volcano Corporation, Rancho Cordova, CA, USA) is 150 cm in total length and has a transverse profile of 3.5 F at the transducer. The nominal transducer center frequency is 20 MHz (free of non-uniform rotational distortion and guidewire artifacts) and it can image a maximum diameter of 20 mm. It can work with 0.014″ or smaller guidewires, and the probe/wire combination can fit in a 6 F guide catheter.

Under fluoroscopic guidance, the IVUS catheter was advanced to the distal segment of the LAD and then withdrawn at a speed of 1 mm/s using a disposable pullback device (Trak Back II, Volcano Corporation, Rancho Cordova, CA, USA) until it came out from the LMCA. The procedure was monitored in real-time using two monitors, one positioned to face the operator and another mounted on the control panel and checked by the US technician. The IVUS data were stored digitally and analyzed offline using specialized arterial analysis software (Volcano s5iTM Imaging System, Volcano Corporation, Rancho Cordova, CA, USA, version 2.5), with all measurements derived from the digitized images converted into millimeters.

#### 2.11.2. Analysis of IVUS Data

Consistent with standard angiographic criteria, the LAD was partitioned into three segments: the proximal segment encompassed the first major septal branch or the first diagonal; the middle segment spanned from the origin of the first diagonal branch to the point and the last diagonal branched off [26,27]. The length of the LMCA, proximal segment, and mid-LAD were quantified using IVUS.

The presence of plaque in each LAD segment was first qualitatively assessed: detecting the atheroma, identifying the tightest stenosis in each segment, and roughly estimating the plaque extension (single or multiple) and its possible encroachment into the lumen, ulceration, dissection, or intraluminal thrombosis. The main quantitative analysis consisted of the following measurements at each of the most narrowed sites in every LAD segment: (1) distance from the LMCA bifurcation; (2) planimetered luminal CSA stenosis: the operator manually placed the initial points along the luminal border of the stenosis and then the whole contour was automatically traced and the area was automatically calculated; (3) the stenotic segment was assessed and a reference segment was chosen in order to planimeter the area; (4) the percentage CSA stenosis was determined as the reference lumen area minus the lesion lumen area divided by the reference lumen area x 00 (Figure 3). The reference segment selected was the most visually normal cross-section within 10 mm proximally to the target lesion but distally to a major side branch; if no proximal reference segment could be identified (e.g., an ostial lesion or diffuse disease, as previously described), a distal reference would be analyzed (also within 10 mm of the target lesion but proximal to a major side branch) [28].

Prior to the diagnostic catheterization procedure, all patients were required to provide written informed consent, in accordance with the guidelines set forth by the Committee for the Protection of Human Subjects at Brigham and Women’s Hospital.

### 2.12. Statistical Analysis

Continuous variables are expressed as mean values ± 1 SD. A *p* value < 0.05 was considered significant. A paired-sample t-test was conducted to compare the maximal velocity and TVI at the stenosis and at the reference site. Quadratic regression was adopted to find the expression of the parabola that best fits the CSA in relation to Asf and transtenotic velocity. A coefficient of determination was assessed. The agreement between quantitative coronary angiography (QCA) and E-Doppler TTE, in terms of the percent cross-sectional area (CSA) of the stenosis, was evaluated using linear regression analysis and the Bland-Altman method [29]. Statistical calculations were performed using IBM SPSS statistics version 23, Armonk, NY: IBM Corp and MedCalc Statistical Software version 19.1.3 (MedCalc Software bv, Ostend, Belgium).

## 3. Results

### 3.1. E-Doppler TTE Data

A localized increase in velocity appeared on Doppler color flow mapping as a localized area of aliased and disturbed signal in all 103 LAD segments studied. The peak diastolic velocity at the stenotic region was significantly higher than that measured at the prestenotic segment both in the significant stenosis (153 ± 64 vs. 36 ± 9 cm/s, mean difference 116 ± 68 cm/s, *p* < 0.0001) and the non-significant stenosis group (52 ± 17 cm/s vs. 32 ± 9, mean difference 19 ± 11 cm/s), with larger differences in significant stenosis patients. The diastolic TVI was also higher at the stenotic segment both in the significant stenosis (0.69 ± 0.34 m) and non-significant stenosis (0.22 ± 0.07 m) subgroups than at the reference site (0.16 ± 0.04 and 0.14 ± 0.03, respectively, *p* < 0.001).

### 3.2. Doppler Performance in Predicting the % Reduction in the Stenotic Area

**Figure 4:**
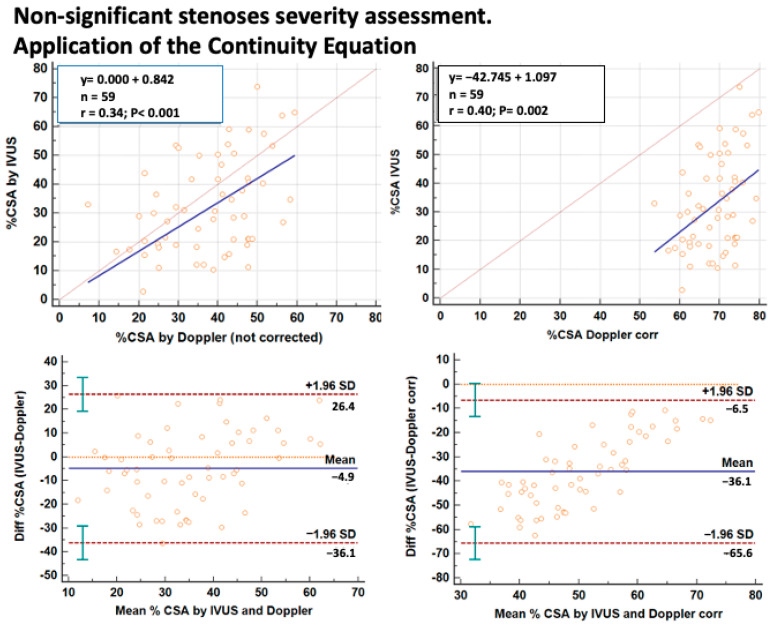
Percent reduction in CSA at the stenosis site in the non-significant stenosis group (59 stenotic LAD segments) revealed by E-Doppler TTE and IVUS. On the left side are the results without correction and on the right are the results with correction of the C-Eq applied to the Doppler method; on each side on the top is a scattergram showing LAD %CSA assessed by the two methods (the blue and the dashed gray lines represent the regression and the identity line respectively); while on the bottom is the plot of the average value against the difference of the IVUS and E-Doppler TTE calculated LAD %CSA (blue line is the mean of the difference and the dashed brown lines the 1.96 standard deviation of the differences with its 95%CI indicated by the vertical green lines); an important overestimation of IVUS CSA by Doppler was ensured in the corrected series. CSA = cross-sectional area; LAD = left anterior descending coronary artery; corr = corrected; C-Eq = continuity equation.

**Figure 5:**
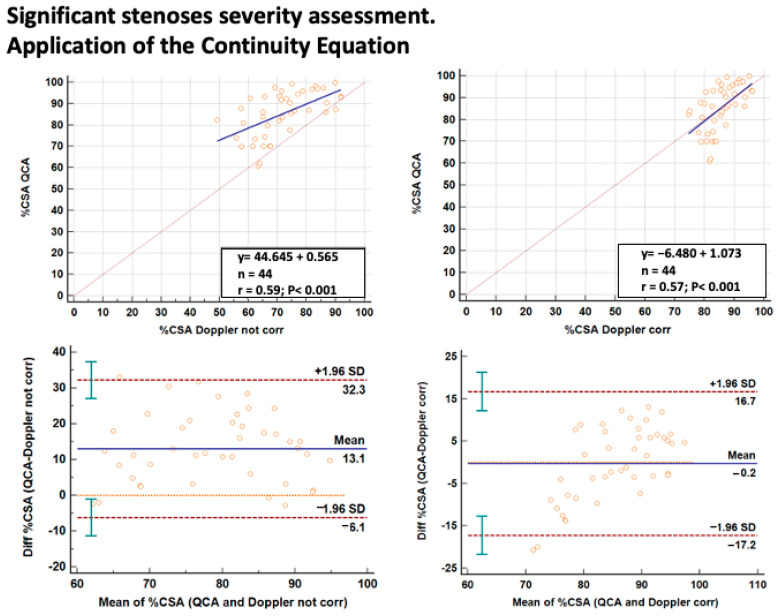
Percent reduction in CSA at the stenosis site in the significant stenosis group (44 stenotic LAD segments) revealed by E-Doppler TTE and QCA. On the left side are the results without correction and on the right are the results with correction of the C-Eq applied to the Doppler method (same layout and legends as Figure 4); CSA = cross-sectional area; LAD = left anterior descending coronary artery; corr = corrected; C-Eq = continuity equation.

The %CSA assessed by Doppler using the C-Eq was correlated with the %CSA as assessed in the significant stenosis group by QCA (86 ± 10%) and in the non-significant stenosis group by IVUS (33 ± 16%). However, it seemed that using the C-Eq formula demanded an opposite approach to correction in the two groups with non-significant and significant stenosis. In particular, despite a good linear relation in the non-significant stenosis subgroup, a systematic error in prediction (overestimation by Doppler) was found when a correction of the C-Eq was adopted for correcting for the difference of the average spatial velocity at the stenotic and the reference site (Figure 4). The opposite occurred in the significant stenosis subgroup where, without correction, the %CSA prediction was not very precise and tended to underestimate the %CSA as assessed by QCA (Figure 5). Therefore, in the significant stenosis subgroup, the correction led to higher precision for the Doppler method.

**Figure 6:**
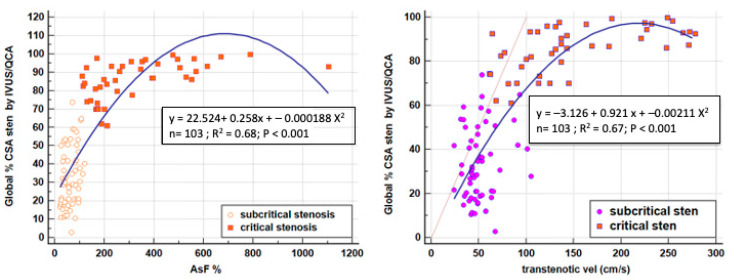
Relationship between the CSA of the stenosis assessed by QCA/IVUS and the AsF (**on the left**) and transtenotic velocity (**on the right**). Both Doppler parameters show a strong quadratic relationship vs. the %CSA (R^2^ = 0.67 and 0.68 respectively, *p* < 0.001). The blue curved lines indicate the quadratic relationship; the straight lines indicate the linear relationship; CSA = cross-sectional area; AsF = accelerated stenotic flow; QCA = quantitative coronary angiography; IVUS = intravascular ultrasounds.

Thus, it was evident that it is crucially important to recognize a non-significant from a significant stenosis before attempting a further grading of the severity by the application of the C-Eq. To address this problem we relied upon the AsF; since an AsF > 122% can successfully predict FFR ≤ 0.08 with a sensitivity and specificity of around 90% [21], we used this cutoff criterion to decide whether to correct the C-Eq formula or not: a value ≥ 122% indicates significant stenosis since more blunting of the velocity profile should be expected at the stenosis site, and correction is warranted; on the contrary, an AsF < 122% indicates mild stenosis with no significant blunting, so no correction is needed for the formula. We confirmed the AsF validity in predicting the stenosis severity, finding a close quadratic relationship (coefficient of determination R^2^ = 0.68, *p* < 0.001) of AsF vs. %CSA stenosis as assessed by QCA and IVUS. This indicates that over a certain value of narrowing (CSA = 80%), there is an exponential increment in AsF (as from an AsF value of 120−150%); therefore, a minimal variation of CSA over that value (imprecisely expressed by the imperfect gold standard we used: the QCA) causes a marked increment in AsF. However, we have to admit that such variation in transtenotic velocity can also be related to the underestimation of maximal transtenotic velocity due to the imperfect insonification of very thin transtenotic jets. The same relationship with CSA is seen with the Doppler parameter of the transtenotic maximal velocity (coefficient of determination R^2^ = 0.67, *p* < 0.001): in this case, the velocity starts to increase exponentially at around 100 cm/s (Figure 6). This further confirms the validity of the transtenotic velocity and the AsF in predicting lumen narrowing and pressure drop through the stenosis during hyperemia (FFR), as previously reported [21].

**Figure 7:**
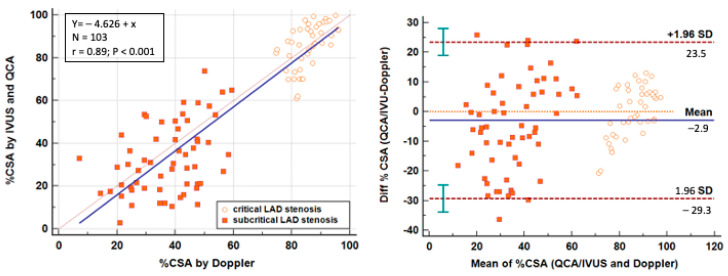
Percent reduction in CSA at the stenosis group in the overall series including 103 LAD stenosis assessed by Doppler (by using the best correcting method of the C-Eq on the basis of the previous results). On the left is a scattergram showing LAD %CSA assessed by the two methods with a strong correlation (r = 0.89, *p* < 0.001), and on the right is the plot of the average value against the difference of the IVUS/QCA and E-Doppler TTE calculated LAD %CSA which shows a minimal bias with limits of agreement within 20% in most cases; the limits of agreement appeared larger in the non-significant stenosis group (abbreviations are the same as those in the previous Figure 4 and Figure 5). Lines explanation like in Figure 4.

Since AsF is a strong predictor of lumen narrowing, it can simultaneously predict the blunting of the velocity profile, indicating when to make the appropriate correction in the formula. By applying the formula of the C-Eq for %CSA measurement using E-Doppler TTE velocity measurements with or without correction based upon AsF, we obtained a strong correlation and sufficient precision for clinical purposes in our global group (both significant and non-significant stenoses). In fact, the correlation coefficient (r = 0.89, *p* < 0.001) was quite high but more importantly, the agreement was good. The bias was minimal but significant (−2.9%, P [H0: Mean = 0] = 0.0311), indicating minimal systematic overestimation by Doppler; the limits of agreement were −29% to 23% with a consistent calculated coefficient of repeatability of 26.8823% (95%CI 23 to 31%). It also appears from the graph that the limits of agreement are tighter for significant than for non-significant stenoses (Figure 7).

## 4. Discussion

We show firstly that the C-Eq can be successfully applied with E-Doppler TTE for %CSA stenosis calculation, confirming sparse previously published data, and secondly, that different handling of the equation (with or without correction) must be done depending on the presence of a critical increment of velocity, as previously validated vs. FFR.

### 4.1. Assessment of Stenosis Severity Using the Continuity Equation

One of the main aspects faced for the first time in this study is that the correction of the shape factor of the spatial velocity profile has to be handled differently in non-significant and significant stenosis, in order to properly apply the C-Eq. This aspect is based on a better knowledge of the factors affecting the spatial velocity profile.

### 4.2. Factors Affecting the Spatial Velocity Profile

The velocity profile is in general affected by three factors [30]. First of all, a greater acceleration of blood adds a flat component to the profile; the more severe the stenosis, the greater the acceleration (as the blood velocity jumps to a very high value in a fraction of a second), and the flatter the profile. It is possible that in mild stenosis, the velocity variation will be so modest that the acceleration is negligible. This aspect could be compounded by the second factor, namely the geometric factors: a converging flow cross-section also flattens the profile, especially in cases of obstructions: the more severe the obstruction, the more effective the flattening component. The last factor is viscosity, which does not apply in our study. Thus, in our study, in the group with significant stenosis the formula with the correction factor provided a good estimate of the severity of lumen narrowing, whereas the non-corrected equation systematically underestimated the severity. Fluid mechanics theory and previously published experimental studies suggest that the cross-sectional velocity profile in a small conduit, such as coronary arteries, is parabolic at a low Reynolds number, but flattens when the velocity increases, and as in a stenosis the flow becomes turbulent [31].

With Doppler, we measured the maximal velocity and not the mean velocity, thus taking into account the true average spatial velocity. Therefore, in cases of flattening (the stenotic site), the measured velocity is close to the mean; instead, in the reference site with a parabolic profile, the mean velocity is half of the maximum: this must therefore be corrected in the formula in cases of flattening at the stenosis [30]. These results are consistent with a previous Doppler study [12] and could be attributed to the existence of distinct velocity profiles in the two sampling regions, as previously demonstrated experimentally [16,30]. While it may not be valid to assume a fully blunted profile (shape factor = 1) at the site of stenosis for all degrees of stenosis severity [16], this assumption remains practical for clinical purposes.

Instead, the %CSA assessment by Doppler was poorly established in the case of non-significant stenosis [14]. We found that the shaping correcting factor was detrimental and a better prediction of CSA was achieved without correction. This is due to the poor acceleration of blood through the stenosis in this range of velocity so that the average spatial velocity does not significantly change in the 2 sites of measurements and no correction is needed. Even though in this subgroup, the limits of agreement with IVUS CSA were fairly large (limits of agreement 26.1–36.4%), they were sufficient for clinical purposes since they were all in the range of mild coronary stenosis, thus allowing meaningful clinical decision-making. The use of AsF (or the maximal velocity), previously validated vs. FFR [21], was crucially important.

### 4.3. Previous Studies

The other approaches proposed for clinically applying the C-Eq as a means of estimating the severity of coronary stenosis are based on an intravascular Doppler flow wire [3,4,5,32,33] and transesophageal Doppler [11,12,34]. However, the first method is limited by the fact that it is invasive and not very feasible in severe grade stenosis; and the second by the fact that it is semi-invasive and can only explore the proximal portion of the LAD.

### 4.4. Clinical Utility of the Application of the Continuity Equation

This non-invasive quantitative approach is attractive for a number of reasons: it is totally non-invasive and, thanks to recent advances, has a very high feasibility close to 100% (100% in our consecutive series of patients selected based on catheterization laboratory logistics); the main factors that have contributed to improving the feasibility are the reduction of HR < 60/min (main trick), the recently described tomographic plane [14,15] and, finally, a sensitive Doppler method (such as the convergent color Doppler mode in the Sequoia equipment or that in GE Vivid 7 equipment) and, in very few cases, the use of contrast enhancement [24]. The stenosis severity can be assessed throughout the LAD (we analyzed proximal, mid, and distal stenoses) and it works over a wide range of stenoses (from mild to severe); this last point has been verified for the first time in this study in terms of the quantitation of the severity. Moreover, the coronary E-Doppler TTE quantitative assessment of the stenosis in the left main and the whole LAD is important in that the approach has got high sensitivity and specificity (around 95%) in coronary stenosis detection that it stands alone in coronary management, especially when associated with coronary flow reserve assessment [24,35].

Finally, blood flow can be consistently recorded in the left circumflex coronary artery (proximal-mid tract and obtuse marginal), as recently demonstrated [15] and in the posterior descending coronary artery [36], possibly extending this quantitation method over the whole epicardial coronary artery system. However, further validation studies are required in this regard [22].

Since the method has high intra-inter observer reproducibility [14], it is ideal for follow-up studies to assess the regression progression, although again, further studies are warranted.

### 4.5. Limitations

Even using the appropriate correction of the formula, the E-Doppler TTE prediction of IVUS/QCA %CSA of stenosis has some limitations despite being clinically valid. For instance, the undetected branches between the reference sampling site and the stenosis may undermine the fundamental assumption on which the equation is based [37]; major correction of the theta angle (>30°) in more tortuous segments may introduce a certain error [30]; and E-Doppler TTE constantly tends to overestimate IVUS/QCA %CSA (bias 2.9%, with the tendency to be larger in the non-significant stenoses), partially because the effective area of flow through the stenosis is somewhat less than the cross-sectional area of the narrowing. This creates the so-called “vena contracta” effect [3]. All these limitations of the Doppler method are compounded by the limitations of the gold standard: the complex geometric characteristic of the stenosis that can affect the functions cannot be summarized simply as the minimal lumen area and diameter, especially in eccentric stenosis with QCA [38,39,40]. Moreover, during IVUS the choice of the reference point within 10 mm from the stenosis to avoid important collaterals is not easy to achieve.

Finally, since a precise evaluation of velocity in two sites using a corrected color-guided pulsed wave Doppler sampling angle is necessary, the procedure can be time-consuming. Nevertheless, the maximal velocity alone can immediately orientate the assessment of the severity of the stenosis.

In very tight stenosis, the jet through the maximal narrowing can be strongly thinned out due to the vena contracta phenomenon. As a result, the insonification can miss the true maximal transtenotic jet. This limitation can partially explain the large range of velocity observed when the stenosis is >90%, as reported in Figure 6.

Coronary computed tomography (CTA) can appear more practical and effective for clinical application than E-Doppler TTE. In reality, CTA is a useful diagnostic tool, but it involves ionizing radiation, which is not only carcinogenic in the long term [41] but may also contribute to the destabilization of the coronary plaques by several mechanisms, such as increasing the permeability of coronary endothelium [42,43] and maintaining inflammation due to Nuclear factor kappa B (NF-κB) [44,45], eventually becoming an important cofactor in causing acute myocardial infarction, sudden death, and angina [46,47]. In addition, CTA is only a morphology-based method and false positives can arise in calcified coronaries [48]; in contrast, E-Doppler TTE is a function-based method that is not affected by calcium and can be integrated by the well-seasoned coronary flow reserve assessment in the distal LAD [49,50,51]. E-Doppler TTE has been shown to be superior to CTA in this respect [52], although larger comparative studies are needed. We have also demonstrated that the lack of a routine specific left circumflex artery (LCX) and right coronary artery (RCA) evaluation is not a problem since atherosclerosis primarily affects the LAD [53]. Using this method, we have shown that a totally normal LAD and LMCA coro flow has a predictive value of absent significant RCA and LCX disease of 97% [54].

## 5. Conclusions

E-Doppler TTE is a feasible and reliable method for assessing the severity of LAD stenosis by applying the C-Eq. For the first time, the application of the continuity equation with transthoracic Doppler has been validated over a full range of stenosis severity in this study.

## Figures and Tables

**Table 1:** Demographic characteristics of examined patients.

	Significant Stenosis	Non-Significant Stenosis
Gender		
Males, #patients (%)	35 (79.5)	41 (82)
Females, #patients (%)	9 (20.5)	9 (18)
Weight, kg	76.29 ± 11.31	79.32 ± 11.74
Height, cm	167.9 ± 9.95	168.66 ± 10.41
Hypertension, #patients (%)	35 (79.5)	39 (78)
Diabetes, #patients (%)	17 (38.6)	17 (34)
Atypical angina, #patients (%)	7 (16)	14 (28)
Typical angina, #patients (%)	20 (45)	11 (22)
Previous MI, #patients (%)	13 (29.5)	12 (24)
Previous PTCA, #patients (%)	11 (25)	14 (28)
LVEF, %	47 ± 22	53 ± 13

*N* = 94 (n significant stenosis group = 44, *n* non-significant stenosis group = 50). MI = myocardial infarction; PTCA = percutaneous coronary angioplasty; LVEF = left ventricular ejection fraction, # = number of patients.

## Data Availability

The data presented in this study are available on request from the corresponding author.

## Supplementary Materials

The following supporting information can be downloaded at: https://www.mdpi.com/article/10.3390/diagnostics13152526/s1.
