# Supplementary material for: Assessment of the Severity of Left Anterior Descending Coronary Artery Stenoses by Enhanced Transthoracic Doppler Echocardiography: Validation of a Method Based on the Continuity Equation
Source: Diagnostics (Basel). 2023 Jul 29;13(15):2526. doi: 10.3390/diagnostics13152526 (PMC10417389; doi:10.3390/diagnostics13152526)
Supplement: Supplementary file 1 [file diagnostics-13-02526-s001.zip › diagnostics-2424324-supplementary.pdf]

Article

Assessment of the Severity of Left Anterior Descending Coronary Artery Stenoses by Enhanced Transthoracic Doppler Echocardiography: Validation of a Method Based on the Continuity Equation

Carlo Caiati, Alessandro Stanca and Mario Erminio Lepera *

Unit of Cardiovascular Diseases, Department of Interdisciplinary Medicine, University of Bari "Aldo Moro", 70121 Bari, Italy; carlo.caiati@uniba.it (C.C.); alessandrostanca@gmail.com (A.S.)
* Correspondence: marioerminio.lepera@uniba.it; Tel.: +39-080-5592-750

Supplementary Materials: Detailed Methods

1. Echocardiographic equipment characteristics and settings.

Echocardiography was performed using an Acuson Sequoia™ ultrasound unit (C256 Echocardiography System, Siemens Healthcare, Erlangen, Germany) and broadband transducer (3V2c). The Color Doppler signal was attained in convergent color Doppler mode at 2.5 or 2.0 MHz transmission frequency, while Spectral Doppler was performed in fundamental mode at 2.5 or 2.0 MHz. The color-coded Doppler setting was adjusted to maximize scanning sensitivity (pulse repetition frequency was set at 16 cm/s [2.5 MHz] or 20 cm/s [2.0 MHz] with minor modulation in special cases, and maximizing the sample volume of color flow mapping) without significantly reducing the frame rate (the color box size was reduced to remain in keeping with a frame rate of >30 Hz). All the studies were digitally stored on the built-in dedicated hard drive.

2. LAD segmentation and anatomy

The proximal portion included: 1) the strictly retropulmonary portion of the vessel extending from the left main coronary artery bifurcation to the plane of the pulmonary valve running horizontally behind the posterior wall of the pulmonary artery (this usually included the first part of the angiographic mid-portion as the first diagonal usually branched off before the pulmonary plane); 2) the mid-LAD portion that is still spatially oriented as the strictly retropulmonary part running beyond the pulmonary valve plane along the left border of the anterior wall of the right outflow tract. The distal segment was the strictly vertically-oriented portion of the interventricular segment.

3. Parasternal windows

The retropulmonary portion was visualised starting from the second intercostal space. Briefly, after obtaining the short axis view of the aorta, the left coronary fossa was identified as the echo-dense region adjacent to the left coronary sinus, delimited by the left pulmonary artery above and the summit of the left ventricle below. It was then attempted to visualise the LAD in this area by slightly angling the transducer up and down, and gradually rotating it clockwise in order to deal with the variable inclination of the vessel in the vertical plane (a 0-90° angle). Once identified in B-mode and then with colour Doppler, the course of the LAD was followed as far as possible.

The approach to the mid-LAD (the upper part of the interventricular portion) has been previously described. Briefly, the upper mid-LAD was visualised using a lower parasternal short axis view of the base of the heart modified by a slight clockwise rotation of the transducer beam, which allows the transection of the upper interventricular portion of the artery running laterally to the right ventricular outflow tract (RVOT) before it

becomes completely vertical (i.e. while it still shares a similar horizontal course to that of the retropulmonary portion).

4. New tomographic plane orientation

Visualisation of the proximal and mid-LAD was substantially improved by moving the transducer as far as possible to the left and exploiting the variable extension of the cardiac notch of the left lung and placing the patient in extreme lateral decubitus. The probe was then angled medially and cranially toward the left coronary fossa and the interventricular sulcus: this approach is much better as it eliminates the interference of lung tissue and situates the LAD in the centre of the sector where ultrasound transmission is optimal. A longer tract of the artery (mainly the conjunction of the retropulmonary and the proximal interventricular portion) is insonified on the same plane with a narrow theta angle, and the diagonal branches are also almost regularly insonified. The approach to the vertically oriented interventricular LAD (i.e. the entire distal portion) has also been previously described.

Apical views were also attempted. Firstly, starting from a 4-chamber view, the probe was angled anteriorly in order to bring the aorta root (oblique slice) into view. A close search for the flow in the left coronary fossa, adjacent to the left side of the aortic root, was attempted by moving the transducer slightly back and forth in order to record blood flow in the LMCA and proximal LAD. Further anterior angling of the transducer allows long-axis insonification of the mid-LAD, which is more vertically oriented and runs along the left border of the right ventricular outflow tract directed slightly toward the right; the transducer was generally moved to an upper intercostal space in order to optimize the mid-LAD window, making it much easier to visualize the blood flow in the diagonal branches.
